# Soccer heading and concussion are not associated with reduced brain volume or cortical thickness

**DOI:** 10.1371/journal.pone.0235609

**Published:** 2020-08-10

**Authors:** Tiago Gil Oliveira, Chloe Ifrah, Roman Fleysher, Michael Stockman, Michael L. Lipton

**Affiliations:** 1 Life and Health Sciences Research Institute (ICVS), School of Medicine, University of Minho, Braga, Portugal; 2 ICVS/3B's—PT Government Associate Laboratory, Braga/Guimarães, Portugal; 3 Gruss Magnetic Resonance Imaging Center, Albert Einstein College of Medicine and Montefiore Medical Center, Bronx, New York, United States of America; 4 Department of Radiology, Albert Einstein College of Medicine and Montefiore Medical Center, Bronx, New York, United States of America; 5 Department of Psychiatry and Behavioral Sciences, Albert Einstein College of Medicine and Montefiore Medical Center, Bronx, New York, United States of America; 6 Dominick P. Purpura Department of Neuroscience, Albert Einstein College of Medicine and Montefiore Medical Center, Bronx, New York, United States of America; University at Buffalo, UNITED STATES

## Abstract

Soccer is the most popular sport in the world and, since it is a contact sport, players are at risk for head injury, including concussion. Here, we proposed to investigate the association of heading and concussion with macroscopic brain structure among adult amateur soccer players. For this study, 375 amateur soccer players (median age 23 years) completed HeadCount-12m to estimate heading over the 12 months prior to MRI and lifetime concussion. T1-weighted 3D magnetization prepared rapid acquisition gradient echo (MP-RAGE) MRI was performed at 3 Tesla. Parcellation was performed using Freesurfer to extract regional gray and white matter volumes as well as regional cortical thickness and total intracranial volume. Regional cortical brain volumes were normalized by total intracranial volume. We categorized heading into quartiles and concussion as 0, 1 or 2 or more. Generalized linear regressions were used to test the association of heading or concussion with each brain morphometry metric, including age and sex, as covariates. Neither heading nor concussion were associated with reduced brain volume or cortical thickness. We observed that greater heading was associated with greater gray matter volume in the left inferior parietal area, which may reflect effects related to training.

## Introduction

Soccer is the most popular sport in the world [1]. As a contact sport, soccer players are at risk for head injury including concussion [2, 3]. Unique to soccer, purposive heading, where the head is used to impact and direct the ball during play, is a fundamental part of the game, which raises concerns for adverse effects of repeated head impacts (RHI) in the context of soccer play. Soccer heading has thus become an area of concern as a potential source of brain injury. Although recognized concussion is more prevalent in American football compared to soccer [4], high levels of heading are associated with concussive symptoms and worse neurocognitive performance (e.g., [5]). In addition, chronic traumatic encephalopathy (CTE) has been reported in former soccer players [6, 7].

Over time, repetitive head impacts, such as occurring with heading in soccer, have the potential to induce long-term structural effects. Indeed, imaging studies have identified adverse microstructural effects of heading, suggestive of traumatic axonal injury [5, 8]. Using diffusion-tensor imaging (DTI) to assess white matter integrity, professional soccer players have been found to exhibit higher radial and axial diffusivity compared to swimmers [8]. Heading has also been shown to be associated with lower fractional anisotropy (FA) in the temporo-occipital region in adult amateur players [5]. Moreover, ventricular enlargement indicative of central cerebral atrophy was shown using CT in a small cohort of former professional soccer players [9]. More recently, using structural magnetic resonance imaging (MRI), it was shown in a study with 15 subjects per group, that former professional soccer players had a steeper age-associated decrease in cortical thickness [10]. Apart from these, we are unaware of other studies that have addressed the potential effect of soccer RHI or concussion on brain macrostructure. The purpose of this study was to determine whether heading or concussion were associated with adverse effects on regional brain volume or cortical thickness in a large cohort of adult amateur soccer players.

## Materials and methods

### Soccer participants

Players were participants in a large longitudinal study of soccer play and its consequences. Players whose data are included in this report were adult amateur soccer players recruited between November 2013 and May 2018 by print and Internet advertisement and through soccer leagues, clubs and colleges in New York City and surrounding areas. Interested individuals were directed to an enrollment website, which, after informed consent, collected screening information. A research team member contacted qualifying individuals, confirmed eligibility, willingness to participate in the study and invited enrollment. Inclusion criteria were: age 18–55; at least 5 years of active amateur soccer play; current active amateur soccer play; at least 6 months of amateur soccer play annually; and English language fluency. Participants were asked to report neurological or other medical diagnoses. Exclusion criteria were: schizophrenia, bipolar disorder; current neurological disorder; pregnancy; or medical contraindication to MRI. At enrollment, each player completed HeadCount-12m (see below), to capture soccer activity, heading and concussion, as well as brain MRI. Details of the overall study design have been published previously [11, 12].

### Exposure measures

HeadCount-12m, part of a suite of web-based assessments that estimate heading over distinct timeframes, was used to generate estimates of heading over the prior 12 months and recognized concussions over the lifetime. Prior publications have reported on HeadCount in detail (e.g., [11–15]). Briefly, participants were asked questions relative to their soccer activity, including the number of months played per year, the mean number of competitive soccer games per week, the mean number of headers per game, the mean number of practices per week, and the mean number of headers per practice. The total number of headers in the past year was estimated by multiplying the mean number of headers in each setting by the number of sessions per week in each setting, converted to month, and then multiplying by the number of months of play per year. Subtotals in each setting were summed to obtain an estimate of total 12-month heading. The HeadCount questionnaire, also asks the number of years that participants have played soccer at a similar frequency and their lifetime concussion history. Participants were instructed to consider a concussion as any head injury for which they sought or were asked to seek medical attention. Due to the high degree of right skew in the exposure measures, we treated each as a categorical variable. Heading was treated as an ordered categorical variable of approximately equal size quartiles and concussion was treated as three categories: “zero”, “1” and “2 or more” concussions over the participant’s lifetime.

### Imaging data acquisition

Whole-brain MR imaging was performed with a 32-channel 3.0-T MR unit (Achieva TX; Philips Medical Systems, Best, the Netherlands) and a 32-channel head coil (Philips Medical Systems, Best, the Netherlands). T1-weighted 3D magnetization prepared rapid acquisition gradient echo (MP-RAGE) was acquired with axial slab selection. Imaging parameters were as follows: TR/TE/TI = 9.9/4.6/900ms, flip angle 8°, 1mm^3^ isotropic resolution, 240 × 188 × 220 matrix and FOV.

### Clinical image review

A board-certified neuroradiologist reviewed all images to detect structural abnormalities and evidence of prior trauma, including microhemorrhage.

### Image processing

Segmentation of brain cortical and subcortical structures from T1-weighted image volumes was performed using the Freesurfer toolkit version 5.3 (https://surfer.nmr.mgh.harvard.edu). This software package implements a semi-automated segmentation workflow including skull removal, normalization of WM intensity, spatial registration to the Talairach standard space and tessellation of gray matter—white matter segmentation. For cortical parcellation, an atlas considering the gyral and sulcal components as separate regions was used [16]. We extracted and analyzed white matter gyral/sulcal volumes, cortical gyral/sulcal volumes, cortical thickness and deep brain structures. Intracranial volume (ICV) was measured for each subject [17]. All volume measures were normalized to intracranial volume (ICV) prior to analysis as follows: Each participant’s ICV was divided by the median ICV of the entire study cohort. Each brain region’s volume was then divided by this normalized ICV.

### Data analysis

Statistical analysis was performed using IBM SPSS software, version 24 (IBM, New York, USA) and GraphPad Prism7 software (http://www.graphpad.com). We constructed separate general linear models to test the association of exposure (heading or concussion) with brain morphometry metrics (volume or cortical thickness) at each location. In each analysis we tested the significance of heading in the 2^nd^, 3^rd^ and 4^th^ quartiles compared to the 1^st^ quartile and of 1 and 2+ concussions compared to 0 concussions. We included sex, age and handedness as covariates. ICV was included as a covariate in analyses of cortical volume, but not of cortical thickness. Bonferroni correction was used to mitigate Type 1 error to a corrected p-value of 0.05 (actual *p* = 0.0002), for 250 corrections. Visualization and representation of brain regions was accomplished using BrainNet Viewer 1.61 [18].

### Participants consent

This study was reviewed and approved by the local institutional review board and complied with the Health Insurance Portability and Accountability Act. Players recruited to participate in the “Einstein Soccer Study”, a multi-faceted longitudinal study of heading and its consequences in adult amateur soccer players, gave written informed consent prior to initiation of study procedures.

## Results

375 amateur soccer players were included in the analysis. Median age was 23 years (mean = 25.7). Average heading for the 12 months preceding MRI was 2188 (median 691; range 0–139561). Players reported 0–6 prior concussions over their lifetimes (median 0, mean 0.71) (Table 1). History of a neurological diagnosis (such as headache or migraine, which were the most frequently reported) or other medical diagnosis was inquired, and due to the small number of participants with any individual medical or neurological history item (Table 1), we were not able to reliably test the effects of specific conditions.

**Table 1:** Demographic characteristics of 365 amateur soccer players.

	Soccer Players (N = 375)	Frequency
**Sex**	Male	70.9%
	Female	29.1%
**Race**	American Indian	0.8%
	Asian	7.2%
	Pacific Islander	1.3%
	Black	16.5%
	White	65.9%
	Chose not to report	7.5%
	Unknown	19.7%
**Education**		
Mean = 15.70	0–13	17.1%
Median = 16	14–15	28%
IQR = 3	16	23.2%
SD = 2.24	17+	31.7%
**Age**		
Mean = 25.67	18–21	36.3%
Median = 23	22–23	16%
IQR = 7	24–27	25%
SD = 7.54	28+	22.7%
**Concussion Count**		
Mean = 0.71	0	63.7%
Median = 0	1	16.3%
IQR = 1	2+	20%
SD = 1.17		
**Heading Count / Year**		
Mean = 2188.22	1	24.8%
Median = 691	2	25.3%
IQR = 1573	3	24.8%
SD = 10072.59	4	25.1%
**ICV**		
Mean = 1395568	0–1273356	24.8%
Median = 1400780	1273357–1400779	25.1%
IQR = 224996	1400780–1498352	25.3%
SD = 166578.78	1498353+	24.8%
**History of neurological diagnosis**	Yes	3.7%
	No	80.5%
	Unknown	15.7%
**History of medical diagnosis**	Yes	9.8%
	No	74.4%
	Unknown	15.7%

All frequencies are reported as percentage of the entire cohort (n = 375). Concussion is reported as 0, 1, 2 or more over the lifetime. Heading is reported as number of heading events during the prior 12 months. IQR: interquartile range; SD: standard deviation; ICV: intracranial volume.

Radiological review of the imaging studies revealed no evidence of prior trauma or other gross structural abnormalities. Thus, no players were excluded due to imaging findings.

**Fig 1:**
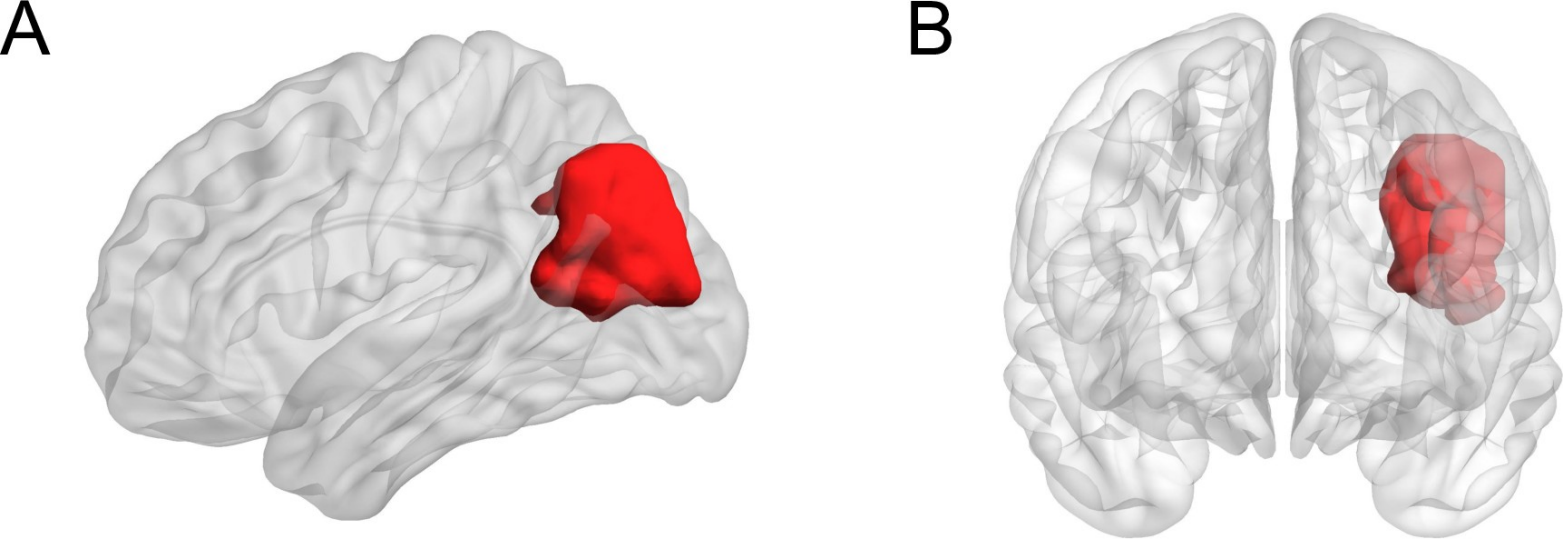
Heading was associated with greater volume of left inferior parietal cortex. (A) Lateral and (B) anterior 3D rendering showing the left inferior parietal cortex in red.

Neither heading, at any level, nor concussion showed a significant association with either lower volume or thinner cortex of any brain region tested. We did find that heading at all levels was significantly associated (*p*<0.0001) with greater volume of the left inferior parietal cortex (Fig 1).

## Discussion

The main finding of this study is that soccer heading or concussion are not associated with lower regional brain volume or cortical thickness in a large cohort of adult amateur players. The diagnosis of CTE in professional and amateur athletes with long histories of sport-related RHI, even in the absence of clinically diagnosed concussion [8], has raised concern regarding the role of RHI in accumulating brain injury and long-term risk for neurodegenerative disease [2]. It is important to consider the largely negative findings of this study of brain macrostructure in context of the nascent study of soccer effects on the brain as well as hypotheses regarding the role of subconcussive RHI and the evolution of brain pathology before gross tissue loss is detectable using volumetry [2].

Several studies have demonstrated microstructural changes related to soccer play and heading [5, 8, 14]. Moreover, studies have shown that subconcussive heading may be associated with concussion-like symptoms and worse cognitive function in the short and longer term [5]. Importantly, similar findings have been reported for professional soccer players [7, 8]. The fact that we do not detect gross structural changes related to amateur soccer RHI thus frames prior microstructural and functional findings as an early and potentially remediable point on a continuum, which precedes overt tissue loss due to frank neurodegeneration. Future work will be essential to better understand the nature of soccer-related RHI, potential for recovery of microstructural and functional effects and how long-term risk varies among individuals.

Heading is a complex motor skill, which requires precisely timed coordination of perception (the trajectory and speed of the incoming ball) and response (e.g., proprioception, muscle contraction). Neuroimaging correlates of neuroplasticity in healthy adults have been reported across a range of training paradigms [19]. Learning to juggle, for example, has been associated with localized gray matter expansion at multiple cortical locations including the mid-temporal area and the left intraparietal sulcus [20] as well as with localized increases in FA on DTI. Long-term practice of a musical instrument, has been associated with greater cortical thickness in the right frontal cortex [21]. In light of these and other examples (reviewed in [19]), we hypothesize that our finding of greater left inferior parietal volume in soccer players who head the ball more may represent an effect of heading skill acquisition. Further work would be required to elucidate pathways and mechanisms subserving the development and maintenance of heading skill. Understanding the potential neuroplastic effects of heading is therefore necessary to inform assessment of the effects of RHI. Moreover, increased levels of heading could be a surrogate marker for time playing soccer and, consequently, higher levels of fitness. Since fitness is associated with greater gray matter volume [22], it may represent an alternate explanation for the higher regional brain volumes we detect associated with heading.

Although we found no lower volume associated with RHI, these findings should be considered in light of several limitations. Even though our sample is the largest ever reported for the adult amateur soccer population, it is nonetheless a subset of that population and may not generalize to other groups such as children. We also studied a relatively young adult population. As such, our findings do not preclude brain volume loss due to neurodegenerative disease in later life. While our localized finding of greater brain volume associated with greater exposure to heading is consistent with a neuroplastic response to skill acquisition, our cross-sectional design precludes any explicit causal inference. We estimated exposure to RHI using HeadCount, which has been validated for this purpose [23]. Nonetheless, the possibility of reporting error or bias cannot be entirely excluded [5, 12]. Additionally, even though the population considered here is comprised predominantly of young adult athletes, we have not acquired data or corrected for body mass index effects, which is another potential limitation. Finally, due to the strong right skew of heading exposure, we treated it as a categorical variable (Table 1). Nonetheless, results were similar when analyses were repeated treating heading as a continuous variable.

In conclusion, we observed no adverse association of soccer-related RHI with brain macrostructure in a large sample of adult amateur players. Further studies and longer follow up will be required to determine whether previously reported adverse effects on brain microstructure and function are associated with such changes over the longer term. On the other hand, the highly localized elevation of brain volume we identified as associated with greater heading suggests a neural correlate for the skill acquisition inherent in heading.
